# Health Science Students’ Perceptions of Hand Hygiene Education and Practice in a South African University: Introducing the University Hand Hygiene Improvement Model

**DOI:** 10.3390/healthcare11182553

**Published:** 2023-09-15

**Authors:** Atheesha Singh, Tobias George Barnard

**Affiliations:** Water and Health Research Centre, Faculty of Health Science, University of Johannesburg, Doornfontein 2028, South Africa; tgbarnard@uj.ac.za

**Keywords:** hand hygiene, nosocomial infection, infection prevention control, University Hand Hygiene Improvement Model

## Abstract

Hand hygiene serves as a critical preventative measure against the spread of acquired infections in healthcare facilities and is an integral component of patient safety programs. While healthcare students in training are typically introduced to the principles underlying hand hygiene, the translation of this understanding into practice is often lacking, and compliance has remained low. This study aimed to evaluate health science students’ in biomedical sciences, chiropractic and emergency medical care, environmental health, complementary medicine, medical imaging and radiation sciences, nursing, optometry, podiatry, and sports and movement studies perceptions regarding hand hygiene education (knowledge and attitude) and practice at a university in South Africa. Consenting students were asked to complete an online questionnaire that tested their knowledge, practices, and skills in handwashing. The ANOVA analysis results suggested significant differences in hand hygiene scores across departments and years of study. The multiple regression analyses confirmed these findings, suggesting that the department of study significantly influenced all aspects of hand hygiene, while the year of study affected hand hygiene skills, and age group influenced hand hygiene practices. Based on these findings, a conceptual model, the University Hand Hygiene Improvement Model (UHHIM), was proposed to enhance the teaching and learning of hand hygiene at the university level. The model underscores the necessity of targeted education, continuous monitoring, and feedback, and the pivotal roles of hand hygiene facilitators and student participation in enhancing hand hygiene behaviors.

## 1. Introduction

The significance of adequate hand hygiene (HH) as an essential tool for the prevention and control of infectious diseases cannot be overemphasized. Despite its simplicity and cost-effectiveness, proper hand hygiene remains suboptimal in many regions worldwide, particularly in developing countries. The World Health Organization (WHO) considers the encouragement of better hand hygiene to be an essential component in the fight against infectious diseases, which makes a substantial contribution to the results of public health [1,2,3,4,5].

However, the realization of this ideal requires more than just access to water and soap; it also requires a comprehensive understanding of the knowledge, attitudes, and practices surrounding hand hygiene. Hand hygiene (HH) is fundamental to the practice of infection prevention and control (IPC) in public, private, and healthcare environments [6,7,8]. According to the WHO, hand hygiene represents a fundamental practice aimed at preventing the transmission of pathogens and reducing the incidence of healthcare-associated infections. It entails the process of cleaning hands with the intent of removing dirt, microorganisms, and other potential contaminants. The practice can be broadly categorized into two main approaches of handwashing with soap and water and hand rubbing through the use of alcohol-based hand rubs (ABHRs) [4,9,10]. It is imperative to note that the proper technique and duration of hand hygiene practices play a pivotal role in their effectiveness. The WHO advocates for a multi-step procedure for both handwashing and the use of ABHRs to ensure comprehensive coverage and maximum efficacy [4,9,10,11,12,13,14].

The hands of healthcare workers (HCW) are the most common vehicles for the spread of healthcare-acquired infections (HAI), as pathogenic microorganisms have been shown to be present on the hands of healthcare workers for 2–60 min following contamination [15,16,17]. Although HH is a simple and effective infection control strategy and an important barrier in the defence against HAI, a non-compliance rate of 40–70% has been reported in healthcare workers [17,18,19,20,21]. In South Africa, it has been reported that at least one in seven patients visiting South African hospitals are at risk of acquiring an HAI due to poor IPC measures, such as improper waste management and poor handwashing techniques [22,23]

Limited information exists on the impact of hand hygiene education and awareness interventions in South Africa, particularly on changing hand hygiene behaviors among health science students [11,12,13,22,23,24]. In previous research, we performed an analytical examination of hand hygiene practices among clinic interns and their patients, utilizing GloGerm™ as a proxy for microbial contaminants, in which we showed that insufficient hand hygiene practices were discernible, based on participants’ hand washing skills after GloGerm™ exposure and their facial expressions and verbal feedback [12]. The study visually demonstrated specific regions of the hands frequently overlooked during washing, shedding light on prevalent practice shortcomings. These insights were instrumental in recommending refinements to hand hygiene protocols within clinic environments. Further research is necessary to inform current and future healthcare workers in the country about the importance of complying with hand hygiene. Studies have found that healthcare providers, including certified healthcare providers and health science students, have low awareness of hand hygiene [25,26].

It is generally agreed that touching anything infectious with one’s hands is the most common way for a person to become infected, particularly in situations where individuals are in close proximity to one another. Among university students, cross-contamination is observed at a notably elevated rate compared to the general population, which can be primarily attributed to the enhanced frequency of interaction between hands and various surfaces [27,28,29,30]. We have highlighted this concern in some of our previous studies and have shown that despite providing university students with hand hygiene education, a significant number of bacterial cells persisted on their hands post-intervention. This discrepancy underscores the need for further exploration of the barriers and facilitators of effective hand hygiene practices within the university setting [11,24,31]. The successful implementation of infection control depends critically on two factors: the method’s capability to decrease the pathogen count and the accessibility of the technique to the involved participants. This underscores the necessity of developing comprehensive strategies that consider these dimensions to successfully combat the spread of infections within educational institutions [4,29,32,33]. Hygiene in reference to hand washing and hand disinfection is frequently misrepresented. Conventional hand hygiene includes washing all surfaces and crevices of the hands with soap and warm water to physically remove dirt and bacteria from the hands [4,10,12,24]. Hand disinfection (rubbing) involves the use of ABHR or sanitizers by rubbing together all surfaces and crevices of the hands to kill or inactivate microorganisms on the surface of the skin, which does not physically remove them [4,5,12,24,29,34,35,36]. Visibly soiled hands must be handwashed to prevent infection and disease [4,5,9,10]. It is important that healthcare workers, students, and staff understand that hand washing with soap and water and hand rubbing with the use of ABHRs are equally important, and that there is a place for both within the patient care arena. To effectively maintain proper hand hygiene and stop the spread of infections, it is important to understand how these two practices differ from one another.

This study aimed to evaluate the knowledge, practices, and skills related to hand hygiene among health science students enrolled at a university in South Africa. This study aimed to identify gaps in awareness, adherence to guidelines, and effective practices and to determine the factors that may contribute to these gaps. The results of this study led to the development of a conceptual model to improve hand hygiene practices through targeted workshops and health promotion interventions.

## 2. Materials and Methods

### 2.1. Study Design and Setting

This cross-sectional, descriptive study was conducted among students from the Faculty of Health Science (FHS) at a university in South Africa during the novel coronavirus disease outbreak in 2020. The FHS comprises the following academic departments: Biomedical Sciences, Chiropractic, Emergency Medical Care, Environmental Health, Complimentary Medicine, Medical Imaging and Radiation Sciences (MIRS), Nursing, Optometry, Podiatry, and Sport and Movement Studies. The study inclusion criteria were comprised of students at any level of study (undergraduate and postgraduate) and registered for the academic year at the time of participation. The questionnaire was anonymized, and participation was voluntary. This faculty was chosen because of the critical role of hand hygiene in health-related disciplines, and the potential impact of improved hand hygiene practices on future professional conduct and patient safety.

### 2.2. Sample Size Estimation

The sample size was calculated using EPI Info version 7.2. 3. 0. With a total population of N = 4109 registered Health Science students at the university, an expected outcome prevalence of 50%, a 95% confidence level, and a 5% degree of error, the sample size (study population) was calculated to be n = 352. A 25% contingency plan to cater for incomplete or declined questionnaires brought the total sample size to n = 439. The questionnaire link was shared with all registered health science students in 2020, and n = 470 responses were received.

### 2.3. Data Collection: Self-Reported Questionnaire

Data were collected through a structured online questionnaire using a method previously published by Ergin and colleagues in 2011 [30], with the authors’ permission. The questionnaire consisted of seven sections containing a study information letter, an informed consent form, and five sections of questions: Section 1: sociodemographic characteristics; Section 2: handwashing frequency; Section 3: handwashing knowledge; Section 4: handwashing practices; and Section 5: handwashing skills. The questionnaire was customized to include department and year of study and a COVID-19-related question on handwashing frequency. The adapted questionnaire was pretested and validated at a research center within the FHS. The data were collected through a Google Forms link and took participants approximately 15 min to complete.

### 2.4. Proposed Model for Hand Hygiene Training

The development of the hand hygiene training model schedule was informed by a two-fold approach. Initially, an extensive literature search was conducted to synthesize practices and guidelines related to hand hygiene [37,38]. This review provided a foundational understanding of the essential elements to be included in the model. This was supplemented with past research from this laboratory, which included pre- and post-test questionnaires, intervention, and simulation (observations), and from hand hygiene studies in the FHS training clinic [11,12,13,31]. The authors have also developed a novel method to quantify bacterial populations on human hands using flow cytometry, which could provide insights into the efficiency of hand hygiene interventions [31]. Subsequently, collected data from this questionnaire-based study contributed to the formulation of the University Hand Hygiene Improvement Model (UHHIM), as it offers empirical information to substantiate the theoretical foundations derived from the literature review [38], ensuring that the model can align with both academic perspectives and student insights. This approach is consistent with best practices in educational research, healthcare, and other fields that seek to develop interventions or models based on both theoretical understanding and empirical evidence.

### 2.5. Data Analysis 

Data were analysed using “IBM Statistical Package for Social Sciences (SPSS)” version 27 and Microsoft Excel 2010. Word cloud was used to highlight the most common responses. Descriptive statistics were used to summarize the data. The analysis of variance (ANOVA) test was used to identify differences in mean scores across different categories of gender, age group, department, and year of study. Multiple regression analyses were performed to ascertain the relationships between these demographic variables and the hand hygiene scores. This study focused on three scales: knowledge, skills, and “self-reported” practice related to hand hygiene. The evaluation involved 12 questions to assess handwashing knowledge, 17 questions to gauge handwashing practice, and 11 questions to estimate handwashing skills. Each participant received one point for each question that they answered correctly on the respective scales according to the criteria of Ergin et al. [30]. To calculate the scores for each scale, the points obtained from all questions with correct answers in that section were summed and divided by the number of questions in that section. The resulting sum is multiplied by 100.

### 2.6. Ethical Consideration

This study followed the ethical requirements of human participant research (REC-667-2020). Before participating in the study, all participants provided informed consent through the online form, and their responses were kept anonymous and confidential.

## 3. Results

### 3.1. Sociodemographic Characteristics

A total of n = 470 student participants took part in the surveys, with n = 464 agreeing to participate. The response rate of 98.7% was mainly attributable to the female students (77.4%). Three categories of age groups were observed, with the majority of participants (49.6%) in the 21–25 age group, followed by 130 (28%) in the 18–20 age group. Each academic department had some students who responded to the questionnaire, with the highest number of responses being observed in the Chiropractic (19.2%), Complementary Medicine (17.9%), and MIRS (15.5%) departments, as shown in Table 1. Most of the students who participated were in their first to fourth year of study, which corresponds to the category of 353 (76.1%) undergraduate students. The study revealed that 213 (45.9%) students lived with their family/partner/spouse, while 145 (41.5%) lived in off-campus residences. Additional demographic data are presented in Table 1.

### 3.2. Handwashing Frequency

**Figure 1:**
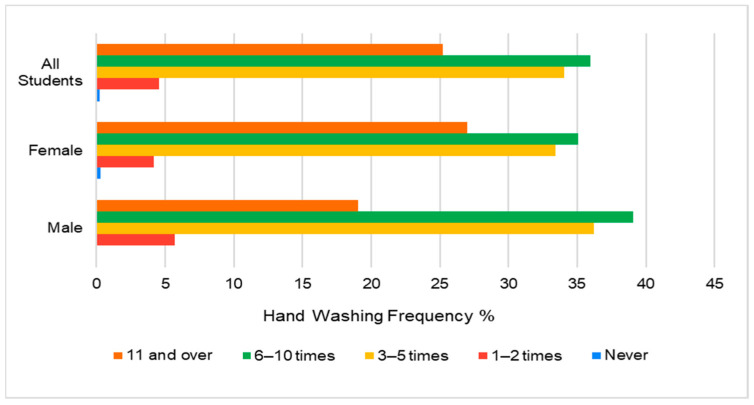
Hand washing frequency between genders (n = 464).

Figure 1 shows the percentage frequency of handwashing among the participants. Overall, 36% and 34% of students washed their hands 6–10 times a day and 3–5 times a day, respectively, while 25% (117) of the students washed their hands 11 times or more. The average “Handwashing Frequency” score was slightly higher for female participants (2.84) than for male participants (2.71), which suggests that on average, females reported washing their hands slightly more frequently than males. Students in the 21–25 and >25 age groups had a similar pattern of handwashing frequency, with the majority washing their hands 11 times or more per day. However, in the 18–20 age group, the majority of students washed their hands 6–10 times per day.

Among female participants, for the question “Do you wash your hands more now due to COVID-19?”, n = 3 said “No”, n = 74 maintained the “Same as Before COVID-19”, and 282 said “Yes”; whereas for the male participants, none said ‘No’, n = 25 maintained the “Same as Before COVID-19”, and n = 80 said “Yes”. In addition, 78.0% of students (362) said that they washed their hands more now due to COVID-19, while 21.3% of students (99) said that they did not wash their hands more due to COVID-19.

**Figure 2:**
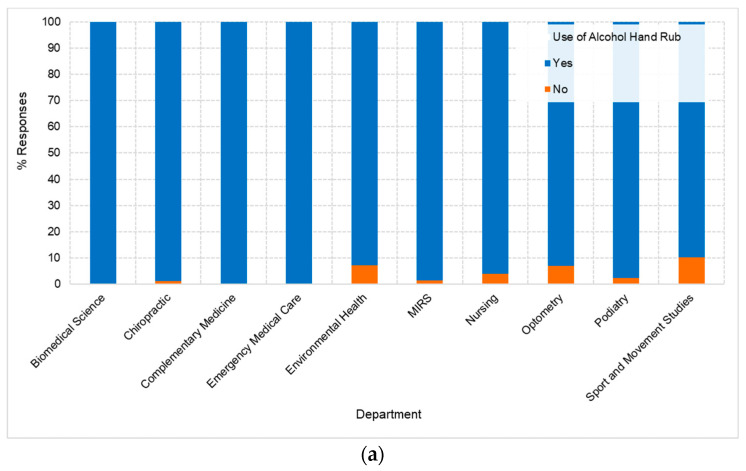

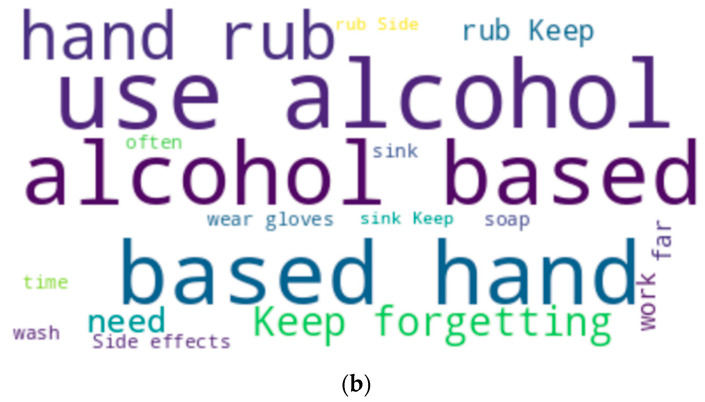
(**a**) Representation of the use of alcohol-based hand rubs or sanitizers across different departments (n = 10). Each bar corresponds to a department, divided into two segments representing ‘Yes’ and ‘No’ responses. (**b**) Word cloud representing the reasons cited by respondents for skipping handwashing. The larger a word appears, the more frequently it was mentioned by participants.

Figure 2a shows variations in the use of alcohol-based hand rubs or sanitizers across departments. Some departments, such as “Biomedical Science”, “Complementary Medicine”, and “Emergency”, showed a higher proportion of “Yes” responses at 100%, indicating the widespread use of alcohol-based hand rubs or sanitizers. In contrast, other departments, such as “Environmental Health”, “Optometry”, and “Sport and Movement Studies”, had variation in the “Yes” responses at 93%, 93%, and 90%, respectively. From the word cloud in Figure 2b, the most commonly cited reason for skipping hand washing is the use of an “alcohol-based hand rub” (n = 290, 62.5%), indicating that respondents often substitute handwashing with using a sanitizer. Another commonly mentioned reason was “forgetting” (n = 136, 29.3%) to wash hands. Interestingly, in this study, n = 30 (6.5%) students indicated that they wore gloves and therefore did not need to wash their hands. Significant differences were found between gender and the reasons for skipping handwashing (*p* < 0.05; *p* = 0.00).

### 3.3. Hand Hygiene Knowledge, Practice, and Skills Responses

**Figure 3:**
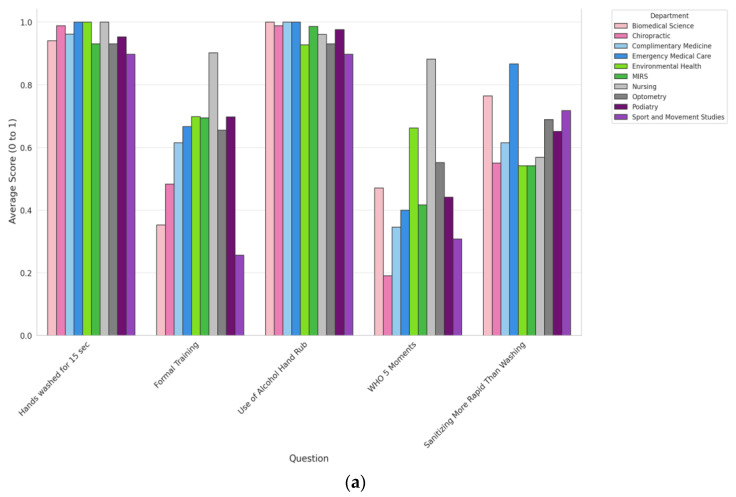

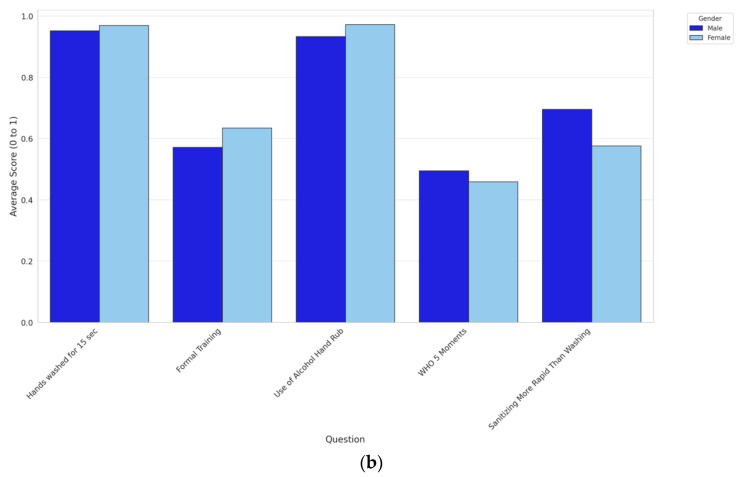
Average scores between departments (**a**) and gender (**b**) for questions related to hand hygiene knowledge on training, guidelines, and the use of alcohol-based hand sanitizers.

Figure 3a illustrates the average scores for each question by department, highlighting the differences in knowledge related to hand hygiene. It is apparent that certain departments exhibit higher average scores for some questions than others. Participants from the nursing department had the highest average score for the “Formal Training” question and the highest average score for awareness on the “WHO 5 Moments” of performing hand hygiene questions. Participants from the Department of Sports and Movement Studies, on the other hand, showed the lowest average scores for most questions.

This variation in participant replies may reflect disparities in the departments’ emphasis on hygiene, curriculum, or the particular requirements of the course of study. Similar to Figure 3a, Figure 3b focuses on gender disparity. Males had slightly higher average scores on the “Hand Sanitizing More Rapidly” question, but females had slightly higher average scores on the “Formal Training” questions. Of the participants, 64% (297) correctly answered that “cold water should not be used for hand washing”, while 420 (90.5%) of the participants correctly responded that “medium hot water” should be used for hand washing. In response to questions about washing the wrists and the back of the hands, 451 (97.2%) and 461 (99.4%) correctly answered “yes”, respectively. Significant differences (*p* < 0.05) were found for responses to “Do you use an alcohol-based hand rub or sanitizer for hand hygiene?” and “Hand sanitizing is more rapid for hand cleansing than handwashing?”

**Figure 4:**
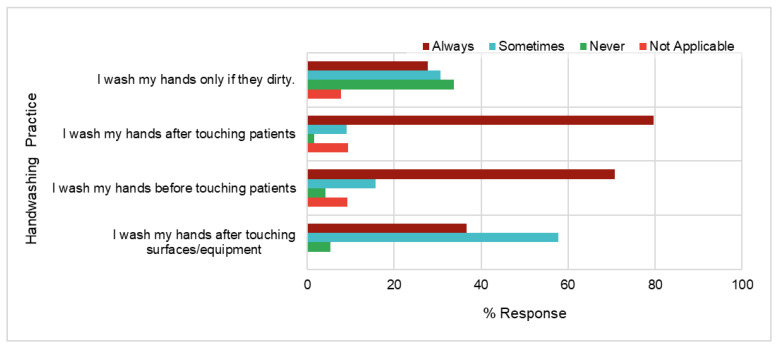
Percentage responses for statements related to hand hygiene practice for all participants (n = 464).

Some of the handwashing practice statements and responses are illustrated in Figure 4. Overal, 34% (157) of the participants selected “never” for the statement “I wash my hands only if they are dirty”, whilst 28% (129) selected “always”. In the study by Ergin et al. [30], the most appropriate response to washing hands only when they are dirty is “never”, and they postulated that healthcare personnel should not limit handwashing to instances when their hands appear soiled. Instead, regular hand hygiene practices are imperative. For the responses to “I wash my hands before and after touching patients”, 71% (328) and 80% (370) of participants correctly answered “yes” and 4% (20) and 2% (8) answered “never”, respectively. Significant differences (*p* < 0.05) were noted for responses between “always” and “never” for all statements shown in Figure 4. Interestingly, 58% (268) of the participants selected “sometimes” for washing their hands after touching surfaces/equipment.

**Figure 5:**
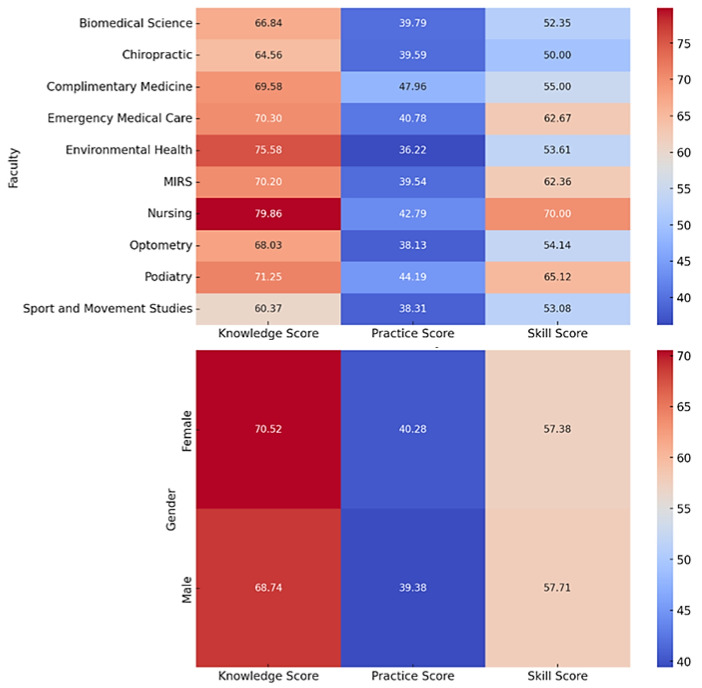
Percentage scores for responses to knowledge, practice, and skills questions according to department and gender (n = 464).

Figure 5 shows the percentage scores for knowledge, skill, and practice according to gender and department. Higher scores are illustrated in shades of red, scaling down to lower scores illustrated in shades of blue. The mean knowledge score is 76.06.% (SD ± 13.00). This implies that the participants had a high level of handwashing knowledge, on average. The practice and skill scores, on the other hand, show considerable variability, with mean scores of 37.22% (SD ± 20.28) and 54.33% (SD ± 21.65), respectively.

The knowledge, practice, and skill R2 values are 0.0328, 0.0117, and 0.0308, respectively. While these scores are statistically significant (*p* < 0.05), they show that demographic variables explain only a small percentage of the variance in the ratings. The outcomes from the ANOVA tests provide several insights. In terms of gender, there is no significant difference in the knowledge, practice, and skill scores, as indicated by *p* > 0.05. As for the age groups, while knowledge and skill scores do not significantly differ across different ages (*p* > 0.05), the practice score with a *p*-value less than 0.05 suggests a notable difference across age groups. Furthermore, departmental differences play a statistically significant role in knowledge, practice, and skill scores, as demonstrated by *p*-values less than 0.05. Finally, when considering the year of study, there is no significant discrepancy in knowledge and practice scores (*p*-values > 0.05), but skill scores show a potential significant difference across different study years, with a *p*-value less than 0.05.

### 3.4. Hand Hygiene Training Model Development

**Figure 6:**
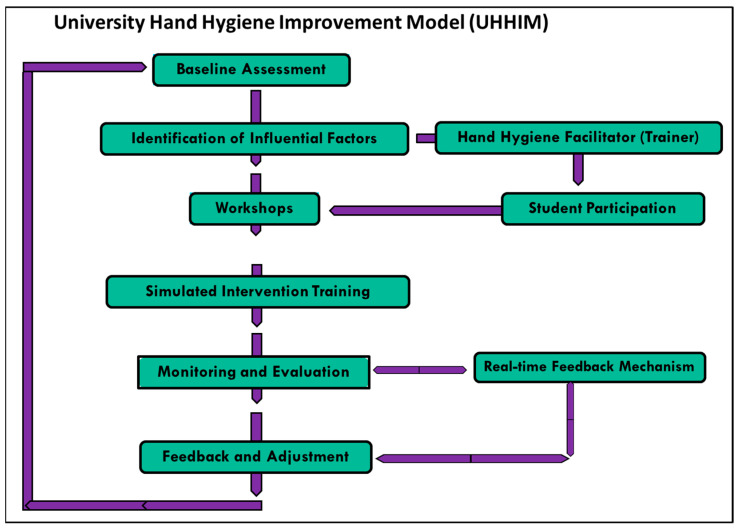
Visual representation of the University Hand Hygiene Improvement Model (UHHIM). The arrows indicate the flow of the process, demonstrating how each stage feeds into the next.

The results obtained underscore the importance of targeted health education and hand hygiene promotion programs to enhance hand hygiene behaviors among university students, particularly those involved in healthcare activities. Building on our findings, it becomes increasingly evident that a one-size-fits-all approach may not suffice in promoting optimal hand hygiene behaviors among health science students. The discrepancies observed across departments suggest that tailored educational and intervention strategies might be more effective. For instance, departments that place less emphasis on hand hygiene could benefit from targeted workshops, seminars, and practical demonstrations. Based on these findings, as well as information from our previous studies and the literature [11,12,13,28,31,34,37,38,39,40,41,42,43,44,45], a University Hand Hygiene Improvement Model (UHHIM) is proposed. The proposed model encapsulates the educational aspects and embeds mechanisms for regular assessment, feedback, and continuous improvement on hand hygiene compliance. Inherent in this model are strategies to bridge the gap between knowledge acquisition and its practical application, ensuring that students not only understand the principles of hand hygiene but are also adept at implementing them in real-world scenarios continuously. The University Hand Hygiene Improvement Model (UHHIM) comprises eight stages, as visually represented in Figure 6. The process begins with a “Baseline Assessment” of the current state of hand hygiene practices, knowledge, and skills among university students, which includes self-reported questionnaires and observation. This is followed by the “Identification of Influential Factors”, which includes hygiene-promoting individuals that can significantly impact hand hygiene behaviors. The Hand Hygiene Facilitator (Trainer) plays a key role in the “Workshops” stage, providing education to students to enhance their hand hygiene knowledge, practices, and skills. Their role is directly linked to “Student Participation”, as the effectiveness of the interventions largely depends on the active engagement and participation of students in the training sessions and other intervention activities. The “Simulated Intervention Training” stage allows students to practice and refine their hand hygiene skills in a safe and controlled environment, preparing them for real-life situations. The training will include scenarios related to preventing nosocomial infections to reinforce the importance of hand hygiene through Glow germ simulation. The effectiveness of these interventions and the changes in hand hygiene behaviors will then be tracked through the “Monitoring and Evaluation” stage. The “Real-Time Feedback Mechanism” stage, integrating technological innovations, and the “Evaluation” stage will further the UHHIM’s reach and efficiency. For instance, smart sensors can be installed in university restrooms to monitor handwashing frequency and send reminders to students. Virtual reality (VR) can be employed in the education phase, offering immersive hand hygiene training sessions. Mobile apps can be developed to provide real-time feedback, reminders, and hand hygiene statistics. Lastly, based on the feedback and results from the evaluation, necessary “Adjustments” are made to the interventions, creating a feedback loop that ensures continuous improvement, refresher training, and observation. This model emphasizes the importance of a systematic and data-driven approach to improving hand hygiene practices in a university setting. The model is cyclic, and each stage is dependent on its precursor, and it can be continuously used to enforce and engage health science students throughout every year of their university training. The ultimate goal is to cultivate an environment where the principles of hand hygiene are seamlessly integrated into the daily routines of healthcare professionals, thereby contributing to enhanced patient safety and reduced transmission of healthcare-associated infections.

## 4. Discussion

This study presents an in-depth investigation into the hand hygiene knowledge and practices of students from ten departments within the Faculty of Health Science (FHS) at a South African university. The inclusion of various academic departments provides a holistic understanding and captures a wide range of nuances, variations, and perspectives on hand hygiene. Different health care disciplines have distinct protocols, patient interactions, and environmental exposures. In a study by Hefzy et al. [46], they showed that healthcare-associated infections are no longer confined to the hospital environment and have been linked to outbreaks within other healthcare environments, such as in a nursing home, rehabilitation facilities, training facilities, outpatient clinics, and the community. The present study found that students’ hand hygiene scores significantly varied across different departments and years of study. Additionally, regression analyses indicated that the department of study considerably influenced all aspects of hand hygiene. It is therefore necessary to study these diverse departments, so as to identify discipline-specific gaps or strengths in hand hygiene practices. Furthermore, the year of study impacted hand hygiene skills, and the age group influenced hand hygiene practice.

When looking at the average results for each gender, the data show that females have fairly better scores for handwashing knowledge and practice, while males have relatively lower scores for handwashing abilities. Ergin and colleagues reported significant gender differences in hand washing scores (*p* < 0.001) [30]. Based on these results, it appears that gender may have a role in determining numerous elements of hand hygiene behavior. However, due to the inherent biases in self-reported data, as well as a lower response rate from males, the findings should be regarded with care.

The mean scores for knowledge, practice, and skills varied throughout the ten departments, with some having greater mean scores than others. The scores for good, moderate, and poor were adapted from [29,30] as follows: ≥70%, good; 50–69%, moderate; and ≤50%, poor. The median knowledge score across all departments ranged from 70 to 79%, showing that most students understood hand hygiene concepts. However, the median practice scores in all disciplines were lower than the knowledge scores, indicating a possible knowledge-practice gap. Nursing had the highest practice and skill levels, whereas Environmental Health had slightly lower values. The median skill scores, such as practice scores, were lower than the knowledge scores. The wide range indicated a significant variance in hand hygiene abilities among departments. This variation could be attributable to the influence of the academic curriculum, variable exposure to health education, or differences in student populations between departments.

The collective results imply that department and year of study may have a considerable influence on hand hygiene competencies, including knowledge, practice, and skill, while age seems to only affect practice. This resonates with previous research findings that showed that health science students’ perceptions and practices of hand hygiene may vary across different fields of study. In addition, the influence of education on handwashing practices has been substantiated by research conducted across various settings, indicating a significant impact (*p* < 0.05) [29,47,48]. This evidence reveals a pattern where senior university students engage in handwashing with greater frequency compared to their junior counterparts [29,48,49]. These findings emphasize the crucial role of university-level hygiene education in reducing the spread of HAI and the need for targeted educational strategies within these institutions to foster enhanced public health protocols. Thus, interventions aimed at improving hand hygiene should consider the unique contexts and requirements of different departments. The study is congruent with previous research indicating that compliance with hand hygiene among healthcare professionals remains low, despite its known efficacy in infection reduction [50,51]. The low compliance rate reported in the study underscores the ongoing challenges in hand hygiene education and practice [41].

The most cited reason for skipping handwashing in this study was the use of an ABHR, followed by a “lack of time”. It can also be suggested that, even with knowledge on hand hygiene, participants were more likely to use the most convenient method for hygiene regardless of its effectiveness. Other reasons, such as “working too far from the sink, forgetting to wash hands, and wearing gloves, therefore not needing to wash hands as often”, were also frequently mentioned. Fewer respondents indicated that they “did not feel the need to wash their hands” or experienced “side effects from soap”. These findings provide important insights into the factors that may influence hand hygiene behaviors. It should be noted, however, that the provided reasons for skipping handwashing are not mutually exclusive, as respondents could select more than one answer. Furthermore, the frequency of each reason does not necessarily reflect its significance or impact on overall hand hygiene practices. Several factors have been reported to negatively impact adherence to recommended practices among HCWs [20,21,22,23,52]. These factors include limited knowledge and awareness, handwashing agents causing discomfort and dryness, inconveniently positioned sinks, a lack of soap and paper towels, time limits, severe workload owing to understaffing, unfamiliarity with rules and practices, and occasional forgetfulness [10,18,20,22,44,52,53,54,55]. A study among Bangladeshi university students showed that student participants skipped hand washing due to “beliefs”, “keep forgetting”, “no need”, and “poor water supply” [48]. In this study, we showed that students use ABHR to clean their hands first unless they are visibly dirty.

Despite understanding the principles of hand hygiene, the practice is not consistently followed, as shown in this study. This finding is supported by other researchers who showed that the lack of consistent adherence to hand hygiene practices among health science students and healthcare professionals is a well-documented issue, despite the benefits [10,29,42,53]. Additionally in previous research in our laboratory, we have demonstrated that “…even with education on hand hygiene, participants were more interested in speeding up the process of hand cleansing and were more likely to use the easiest/most convenient method of cleansing regardless of its effectiveness…” [11]; “mandatory hand hygiene protocol demonstration should be done quarterly or each semester…” [13]; and “… student interns could see the residue of GloGerm^TM^ fluoresce under the black light. This form of simulation used was successful as it showed interns that even if they believe they are washing their hands properly, they are not…” [12]. To bridge this gap, we introduced the University Hand Hygiene Improvement Model (UHHIM), as shown in Figure 6. This model operates on the premise that hand hygiene behaviors can be improved through targeted education and continuous monitoring and feedback, emphasizing the importance of considering demographic and other influential factors when developing and implementing hand hygiene interventions. The literature confirms that education and training improve hand hygiene compliance [40,41,43,56]. Moreover, continuous monitoring and feedback have been found to be effective strategies to enhance hand hygiene adherence [40]. In past research in our laboratory [19,21], we have found that while training and intervention improved student participant knowledge and attitudes about hand hygiene, their actual practices, as evidenced by microbial data from their hands, did not align with their knowledge and attitudes. We deduced that the presence of bacteria on the hands could be a result of inadequate hand hygiene practice rather than a lack of hand hygiene frequency. According to Nieva and Sorra [57], training approaches to enhance hand hygiene among health science students should incorporate evaluation and scenario-based learning, as well as requiring role models and a shift in infection control culture for sustainability. The UHHIM model focuses on the need for specific education, ongoing monitoring, and regular feedback. It also emphasizes the important roles of facilitators (trainers) who guide hand hygiene practices and the active participation of students in improving hand hygiene habits. By emphasizing targeted education, continuous monitoring, and feedback, the model could enhance the teaching and learning of hand hygiene at the university level [58,59]. The inclusion of hand hygiene facilitators and student participation in the model also reflects the importance of interactive learning and student involvement in behavior change [60,61]. The model should be adapted to align with students’ individual and cultural beliefs, departmental influences, and educational progression, offering a personalized approach to future implementation. The targeted education will involve department-specific or year-of-study-specific programs that cater to the unique needs and challenges of different student groups. Continuous monitoring and feedback could provide students with regular updates on their hand hygiene performance, thus motivating them to improve. Additionally, continuously evaluating the effectiveness of the training model using both quantitative (e.g., hand hygiene compliance rates) and qualitative (e.g., feedback from participants) methods will address emerging challenges or new information. Continuous efforts and promotion strategies are required to maintain acceptable levels of adherence to the practice of hand hygiene [59]. The importance of hand hygiene transcends the boundaries of universities and becomes paramount in hospitals, clinics, and other health facilities where the stakes are significantly higher. The foundation laid during academic training sets the tone for future professional conduct. Thus, universities and health training institutions have a pivotal role in not only imparting knowledge but also fostering a culture of rigorous hand hygiene practices. Moreover, interdepartmental collaborations could be a novel approach to standardize hand hygiene education across diverse courses. Sharing best practices, resources, and expertise might lead to a more holistic and unified approach to hand hygiene training. Given the global emphasis on infection prevention, especially in the wake of pandemics, integrating technology and innovation into hand hygiene education might be the way forward. Virtual reality simulations, interactive apps, and real-time feedback systems can engage students more effectively, making the learning process more dynamic and impactful. Lastly, it is crucial to emphasize continuous learning and adaptation. As new pathogens emerge and healthcare environments evolve, hand hygiene protocols and education must keep pace. Regular curriculum reviews, stakeholder feedback, and staying abreast with global best practices will ensure that our approach to hand hygiene remains relevant, effective, and aligned with the overarching goal of enhancing public health and safety.

The UHHIM is based on theories such as the Health Belief Model and the Theory of Planned Behavior [62,63,64]. These theories suggest that people’s behaviors, including hand hygiene habits, are influenced by their knowledge, views, attitudes, and social and environmental context. A study in Benin on healthcare workers pointed out the need for better hand hygiene in hospitals, especially in developing countries [39,44]. The study recommended support from the organization and continuous education for everyone in the hospital to bring about a significant change in the healthcare system. The COVID-19 pandemic has shed more light on the fundamental need of maintaining proper hand hygiene in the fight against the transmission of infectious illnesses [65]. This global health catastrophe, according to the World Health Organization, highlighted the urgent need for effective models to enhance hand hygiene habits, not just in healthcare settings but also in the broader population. These models are needed both in healthcare settings and in the wider society [9].

Zheng and colleagues showed that the underlying mechanism of hand hygiene behavior among HCWs is based on the capability, opportunity, and motivation-behavior model. Their study found that to improve hand hygiene behavior, more efforts should focus on resource provision and hand hygiene motivation enhancement [39,66]. Therefore, to improve hand hygiene habits, we need strategies that not only increase knowledge but also positively affect attitudes and views. These strategies should also create an environment that supports good hand hygiene practices.

The UHHIM model emphasizes the importance of practical, hands-on training in improving hand hygiene behaviors and aligns hand hygiene education more closely with the real-world context of preventing nosocomial infections in healthcare settings. By incorporating workshops and simulated intervention training, healthcare professionals and trainers can actively engage in practical exercises that simulate real-world scenarios. This approach allows for a more effective assessment of hand hygiene behaviors and enables necessary adjustments to be made based on feedback and results. Ultimately, the UHHIM model aims to bridge the gap between theoretical knowledge and practical application, ensuring that hand hygiene education is tailored to the specific challenges faced in healthcare settings.

## 5. Conclusions

The present investigation into the hand hygiene practices of health science students at a South African university has unveiled significant disparities, with demographic variables surfacing as crucial determinants of hand hygiene behaviors. A prominent factor influencing these behaviors is the department of study, highlighting potential variations in hand hygiene emphasis across distinct courses. Additionally, age and year of study appear to indicate a progression in awareness and experience over time. However, it is salient to note that the results from this study account for a marginal proportion of the variance in hand hygiene scores. This suggests that there may be other unmeasured factors influencing hand hygiene behaviors. The limitations of the study include the reliance on self-reported data, a scarcity of male participants, and underrepresented age groups, which could potentially introduce biases in the findings. Given these insights, subsequent research endeavors should focus on uncovering additional determinants of hand hygiene behavior, encapsulating both individual and institutional perspectives. The emergence of these gaps underscores the pressing need for a structured approach to hand hygiene education and practice. To this end, the University Hand Hygiene Improvement Model (UHHIM) has been proposed. The model emphasizes the imperative of targeted education, sustained monitoring, and timely feedback, placing significant importance on the roles of hand hygiene facilitators and proactive student participation. The results from all our research on hand hygiene underscore the imperative of targeted interventions to bolster hand hygiene. These interventions should be especially honed towards specific departments and tailored for younger and less experienced students in health training. Through the integration of the UHHIM, it is anticipated that the university can bridge the identified gaps in awareness, adherence, and effective practices, ultimately fostering a culture of optimal hand hygiene among its health science students. To gain a deeper understanding of the evolution of students’ hand hygiene perceptions and practices, future studies could adopt a longitudinal approach, tracking the same cohort over multiple years.

## Figures and Tables

**Table 1:** Sociodemographic profile of health science students.

Variables	(n = 464) Frequency (%)
Gender	
Female	359 (77.4)
Male	105 (22.6)
Age (Years)	
18–20	130 (28.0)
21–25	230 (49.6)
>25	104 (22.4)
Academic Department	
1. Biomedical Science	17 (3.7)
2. Chiropractic	89 (19.2)
3. Complimentary Medicine	83 (17.9)
4. Emergency Medical Care	26 (5.6)
5. Environmental Health	15 (3.2)
6. Medical Imaging and Radiation (MIRS)	72 (15.5)
7. Nursing	51 (11.0)
8. Optometry	29 (6.3)
9. Podiatry	43 (9.3)
10. Sport and Movement Studies	39 (8.4)
Year of Study	
1st	99 (21.3)
2nd	118 (25.4)
3rd	114 (24.6)
4th	81 (17.5)
5th	25 (5.4)
6th	21 (4.5)
7th	0 (0)
8th	6 (1.3)
Student Category	
Undergraduate	353 (76.1)
Postgraduate	111 (23.9)
Living Location	
Campus Residence On Site	32 (6.9)
Campus Residence Off Site	145 (31.3)
With Family/Partner/Spouse	213 (45.9)
Commune	43 (9.3)
Alone	31 (6.7)

## Data Availability

The data presented in this study are available in Supplementary Materials.

## Supplementary Materials

The following supporting information can be downloaded at: https://www.mdpi.com/article/10.3390/healthcare11182553/s1. Table S1: Data on Hand Hygiene Knowledge Attitude and Practices.
